# Combined analysis of mRNA–miRNA from testis tissue in Tibetan sheep with different FecB genotypes

**DOI:** 10.1515/biol-2022-0605

**Published:** 2023-05-23

**Authors:** Wu Sun, Shike Ma, Xiayang Jin, Yuhong Ma

**Affiliations:** Department of Animal Science and Veterinary Medicine, Qinghai University, Xining, Qinghai, 810016, China; Key Laboratory of Livestock and Poultry Genetics and Breeding on the Qinghai-Tibet Plateau, Ministry of Agriculture and Rural Affairs, Xining, 810016, China; Plateau Livestock Genetic Resources Protection and Innovative Utilization Key Laboratory of Qinghai Province, Xining, 810016, China

**Keywords:** mRNA-seq, miRNA-seq, reproduction, network, candidate genes

## Abstract

Testis size is important for identifying breeding animals with adequate sperm production. The aim of this study was to survey the expression profile of mRNA and miRNA in testis tissue from rams carrying different FecB genotypes, including the wild-type and heterozygous genotypes in Tibetan sheep. Comparative transcriptome profiles for ovine testes were established for wild-type and heterozygote Tibetan sheep by next-generation sequencing. RNA-seq results identified 3,910 (2,034 up- and 1,876 downregulated) differentially expressed (DE) genes and 243 (158 up- and 85 downregulated) DE microRNAs (miRNAs) in wild-type vs heterozygote sheep, respectively. Combined analysis of mRNA-seq and miRNA-seq revealed that 20 miRNAs interacted with 48 true DE target genes in wild-type testes compared to heterozygous genotype testes. These results provide evidence for a functional series of genes operating in Tibetan sheep testis. In addition, quantitative real-time PCR analysis showed that the expression trends of randomly selected DE genes in testis tissues from different genotypes were consistent with high-throughput sequencing results.

## 1 Introduction

In mammals, testes are male reproductive organs that produce sperm, secrete androgenic hormones, and maintain male characteristics [1]. In production, we usually use a scrotal circumference as a proxy for testicular size, which is significantly and positively correlated with testicular size, thus indirectly indicating that the trait is easy to measure and has high heritability [2]. Studies have found positive correlations between testicular size and sperm motility, production, concentration, and ejaculate volume and a negative association with abnormal sperm percentage in livestock animals, including cattle, sheep, goat, and pig [3,4,5,6]. Testis size affects the age at puberty and the postpartum interval of offspring in females [7,8]. Therefore, the selection of elite sires based on testicular size is an effective method to improve reproductive performances. Thus, the testes are one of the important indicators for predicting male reproductive ability.

Testicles descend before puberty is reached and grow in size after puberty, increasing in volume by more than 500% [9]. This growth is due to lytic function, sperm production, and interstitial fluid and Sertoli cell fluid production. Sertoli cells and germ cells rapidly proliferate at birth and undergo a radical change in morphology and function at the onset of puberty [9]. This is due to a complex biological process, involving genetic and microRNA modulation, which is essential for testis development and sperm function in adult males. A gene called *INHBA* has been shown to affect testis morphogenesis, testicular cell proliferation, and testis weight in mice, and has a potential role in regulating testis size [10,11,12]. Among the regulators of spermatogenesis, small RNAs, including microRNAs (miRNAs) and PIWI-interacting RNAs (piRNAs), have recently attracted great attention [13]. miRNAs are small (∼22 nucleotides) endogenous RNAs that negatively regulate gene expression by targeting the 3′-untranslated region (3′-UTR) [14] or coding region [15] of mRNAs. Studies have shown that miRNAs play an important role in testicular development and spermatogenesis in mammals. New generation sequencing tools on mouse testis tissues at 8, 16, and 24 d found key miRNAs such as miR-34c, miR-221, miR-222, miR-20, and miR-106a [16]. Research has shown that miR-34c may inhibit the proliferation of male reproductive stem cells in dairy goats, and differentially expressed (DE) mRNAs and miRNAs have been identified in pig testis tissues at different developmental stages [17]. Expression profile and biological function studies on testicular tissues of yak, cattle, and cattle-yak have found that miRNAs are involved in apoptosis and are associated with the blockade of cattle-yak spermatogenesis [18]. Another well-known gene that controls reproductive traits in sheep, *BMPR*-*IB* (FecB), has been studied by many researchers, focusing on ewes [19,20,21]. Ewes carrying the heterozygous genotype tend to produce more lambs, and, conversely, individuals carrying the wild type produce fewer lambs [22]. The relationship between ram fertility and FecB has not been elucidated, and we propose that a molecular mechanism exists for the higher fertility of rams carrying the heterozygotes compared to wild-type individuals. Although these studies describe mRNA and miRNA expression profiles of testicular tissues from different livestock animals, the genetic mechanisms of the testis tissue from different genotypes on FecB locus have not been investigated. Studies on the expression profiles of mRNA and miRNA in testis tissue of rams carrying wild type and heterozygotes in Tibetan sheep are lacking.

An indigenous sheep breed (Tibetan sheep) was selected for this study. This sheep is widely distributed as livestock in the Qinghai-Tibet Plateau (QTP) and adjacent areas in China. Tibetan sheep are well adapted to the harsh natural environment and are irreplaceable livestock in the high-altitude areas of QTP. They are the main source of meat in Tibet and have been found to have a low rate of double birth. Qiao et al. found FecB mutations in the ewes of Tibetan sheep [23,24]. Using a molecular marker of the FecB gene, the core population of multiparous Tibetan sheep was established, and the technical problems of low fecundity in ewe were solved. However, ram fertility is poorly researched. Mounting evidence indicates a series of genes that are critical for testis development, especially testis size and volume. *AKT* was shown to act as an anti-apoptosis gene in animal growth development and plays an important role in mouse testicular spermatogenesis [25]. Moreover, *PLK1* was shown to be critical for testis development, and *EHD4* is required for the survival of germ cells and attainment of normal prepubertal testicular size in mice [26]. *EPAS1* is required for spermatogenesis in postnatal mouse testes. Postnatal *EPAS1* ablation leads to male infertility, with reduced testis size and weight [27]. *KITl* is crucial for the testicular environment postnatally, and *KIT* is important for the proliferation and survival of spermatogonia [28].

Tibetan sheep reach sexual maturity at 8 months. In our preliminary research, we made a breakthrough in identifying the presence of FecB in Tibetan sheep and using this molecular marker for selection to expand the core population of Tibetan sheep. Because the relationship between ram fertility and FecB has not been elucidated in Tibetan sheep, we propose a potential molecular mechanism for the higher fertility of rams carrying the heterozygotes compared to the wild type. FecB has been reported more frequently in ewes and rarely in rams. This study is the first to study the relationship between FecB and testicular transcriptome in rams. In this study, we used Solexa deep sequencing to investigate mRNA, miRNA profiles, and construct mRNA–miRNA networks for testes from Tibetan sheep with different genotypes. This work provides a theoretical basis for the study of ram fertility and provides insights into testicular development in ruminants.

## 2 Materials and methods

### 2.1 Animals and sample collection

Blood from 40 individual rams was randomly tested from the Tibetan sheep population for genotyping at the FecB gene locus (A746G) according to molecular detection methods for the major gene FecB in sheep [29]. In general, there are three genotypes at the FecB mutation locus: AA (also known as ++ or wild type), AG (heterozygous), and GG (homozygous). Based on the genotyping results of the FecB locus and the preciousness of heterozygote rams, which can be irreversibly damaged by slaughter, we selected six rams, including three wild types and three heterozygotes, for high-throughput sequencing. To ensure that the environmental conditions were similar throughout the experiment, all the lambs were housed in a well-ventilated room with controlled temperature and humidity. Testicular tissues were collected as described by Xu et al. [30]. All the animals and samples used in this study were collected according to the protocol approved by the institutional animal care and use committee of Qinghai University.


**Ethical approval:** The research related to animal use has been complied with all the relevant national regulations and institutional policies for the care and use of animals, and has been approved by the institutional animal care and use committee of Qinghai University.

### 2.2 RNA isolation and quantification

A total amount of 1 μg RNA per sample was used as input material for the RNA sample preparations. The total RNA and small RNA were isolated using TRIzol® reagent (TransGen Biotech, Beijing, China). RNA purity was checked using a NanoPhotometer spectrophotometer (IMPLEN, CA, USA). RNA degradation and contamination were monitored on 1% agarose gel. RNA concentration and integrity were measured using Qubit^®^ RNA Assay Kit in Qubit^®^ 2.0 Fluorometer (Life Technologies, CA, USA) and RNA Nano 6000Assy Kit of the Bioanalyzer 2100 system (Agilent Technologies, CA, USA), respectively. The experimental protocols were performed according to the manufacturer’s technical instructions.

### 2.3 mRNA sequencing and analysis

cDNA libraries were prepared by first isolating total RNA from testis tissue. Total RNA was then reverse transcribed into cDNA, which was then amplified to create cDNA molecules. cDNA libraries were used for transcriptome sequencing. In this study, six RNA libraries were constructed and named TWT1, TWT2, TWT3, THT1, THT2, and THT3. Sequencing libraries were generated using the NEBNext Ultra RNA Library Prep Kit for Illumina (NEB, USA) following the manufacturer’s recommendations. After cluster generation, the libraries were sequenced on an Illumina HiSeq Xten platform and 150 bp-paired end reads were generated. Clean reads were obtained by filtering out adapter sequences and removing low-quality reads from raw data. Simultaneously, Q30 and GC content of clean data were calculated. RNA-seq reads were aligned to the sheep reference genome (Oar_v3.1) using Tophat 2.0.10 [31] with default parameters. The gene expression level was calculated by reads per kilo-base per million reads after the read numbers mapped to each gene were counted by HTSeq v0.6.1 [32]. DE genes (DEGs) were examined using the DEGseq R package (1.12.0) [33]. A corrected *P*-value of 0.05 and an absolute value of log2 (fold change) of 1.5 were set as the thresholds for significant differential expression. Gene Ontology (GO) enrichment analysis of the DEGs was implemented by the GOseq R packages based on Wallenius non-central hyper-geometric distribution [34].

### 2.4 miRNA sequencing and analysis

miRNA libraries were prepared by first isolating total RNA from testis tissue. Total RNA was processed with a miRNA-specific reverse transcription step to create a miRNA library. miRNA libraries were used for miRNA expression profiling. Six small RNA libraries were constructed: SRWT1, SRWT2, SRWT3, SRHT1, SRHT2, and SRHT3. Sequencing libraries were generated using NEBNext^®^ Multiplex small RNA Library Prep Set for Illumina^®^ (NEB, USA) following the manufacturer’s recommendations, and index codes were added to attribute sequences to each sample. Small RNAs ligated with 50 and 30 adapters were reverse transcribed and amplified. Library preparations were sequenced on the Illumina HiSeq Xten platform according to the manufacturer’s instructions. After removing adapter sequences, reads containing poly-N, and low-quality reads, the clean reads were mapped to the Repeatmasker and Rfam database to remove tags originating from repeated sequences, rRNA, tRNA, snRNA, and snoRNA. The miRNA expression levels were estimated by transcript per million values. Differential expression analysis of miRNA was performed using the DEGseq R package [33]. *P*-value was adjusted using *q*-value, and *q*-value < 0.01 and |log2 (fold change)| > 1 were set as the threshold for significantly differential expression by default.

### 2.5 Construction of an mRNA–miRNA regulatory network in testicular tissue of different genotypes

miRNAs can be incompletely complementary base-paired to the target mRNA to inhibit its expression. Spermatogenesis is subject to various factors: several miRNAs and their target genes are involved in the regulation of this complex process. True DE target genes (TDETGs) came as a result of the overlap of the target genes of DE miRNAs with the DEGs. The methods to construct mRNA–miRNA networks consist of three key points. First, data collection was performed by collecting DE miRNA and mRNA data from both the wild-type and heterozygote groups. Second, data analysis was performed using RNAhybrid tools [35] to compare the data and identify miRNA and mRNA sequences that are DE in the same tissues. Third, network construction was performed using Cytoscape software [36] to create a network diagram with DE miRNA and mRNA nodes.

### 2.6 Validation of mRNA by qRT-PCR

Total RNA was extracted using TRIzol^®^ reagent (TransGen Biotech, Beijing, China) as described by the manufacturer. After acquiring high-quality total RNA, mRNAs were reverse transcribed using the RevertAid™ First Strand cDNA Synthesis Kit (TransGen Biotech, Beijing, China). Quantitative real-time PCR (qRT-PCR) analyses on the mRNAs were performed using SYBR Green PCR Master Mix (ABI, USA, 4304437) in a Bio-rad CFX96 real-time system. Beta actin was used as an internal control for each mRNA. Primer sequences of all mRNAs were designed using primer 5 (Table 1). For mRNA quantification, the reaction conditions were: 95°C for 10 min, followed by 40 cycles of 95°C for 10 s and 59°C for 50 s. Relative mRNA expression was evaluated using the 2^−△△CT^ method [37]. Differences were considered significant when *P* < 0.05 using one-way analysis of variance. All statistical analyses were performed using general linear models in R. Visualization of graphs was done with the ggplot package [38] in R.

**Table 1:** mRNA primers for reverse-transcriptase quantitative PCR for different genotype testis tissue

Gene name	Accession numbers	Primer sequence (5″ → 3″)		Product size (bp)	Annealing temperature (°C)
*AKT*	NM_001161857	Forward	CGCCGATGATCGATAACCCT	156	60
		Reverse	CAGCGGTCTGCTACTTCCTT		
*EHD*4	XM_004010473	Forward	TGGCAGTTTTGGTGGTATGC	219	60
		Reverse	CCTTTATTCCTCCTGGAGCCC		
*TGFBR*3	XM_042250942	Forward	TGTGGTTGGTTCGATGCAGA	224	60
		Reverse	CTCCTCCCCGTGTTTAGCAG		
*ODF*3	XM_004019747	Forward	TTTCTGCCCGAACCAAGACC	176	60
		Reverse	CACGTCAGTCTGGTGGTAGG		
		Reverse	CCATGTCTCGGTAGCACTGG		
*INHA*	NM_001308579	Forward	AGAGCCGCCCTCAATATCTC	146	60
		Reverse	GGTTGGGCACCATCTCATACT		
*VIM*	XM_004014247	Forward	GGGACAAGATAGCTACCCGC	260	60
		Reverse	GTCGAAGGCCCGTGTAAAGA		
*COL1A*1	XM_027974705	Forward	GGTGTCGGCGGGTTGTC	289	60
		Reverse	TTGTACGCCCCCAGAATTG		
*COL1A*2	XM_004007726	Forward	TGGACCAATGGGGTTGATGG	272	60
		Reverse	CCCTAATGCCCTTGAAGCCA		
*ACTB*	NM_001009784	Forward	CCTGCGGCATTCACGAA	134	60
		Reverse	GCGGATGTCGACGTCACA		

## 3 Results

### 3.1 mRNA expression analysis from testes in different genotypes

To obtain differential comparative transcriptome profiles for testes of different genotypes, six libraries, namely TWT1, TWT2, TWT3, THT1, THT2, and THT3, were constructed and sequenced by Illumina HiSeq Xten platform. The major characteristics of the six libraries are summarized in Table 2. The Q30 values of the six libraries were >88.47%, and GC content was ∼52.49%. Thus, sequencing quality was feasible. Approximately 65.68–74.72% of clean reads were mapped to the sheep reference genome (Oar_v3.1), of which 63.27–73.03% were uniquely mapped, whereas 1.70–2.62% showed multiple matches. Finally, 2,034 up- and 1,876 downregulated genes were identified in the wild-type group compared to the heterozygote group. These genes may be regulators of testicular growth and spermatogenesis.

**Table 2:** Basic characteristics of mRNA-seq

Sample	TWT1	TWT2	TWT3	THT 1	THT 2	THT 3
Group	Wide type	Wide type	Wide type	Heterozygote	Heterozygote	Heterozygote
Raw reads	20,588,268	22,046,944	22,051,621	21,648,924	21,719,049	28,164,621
Clean reads	20,577,992	22,035,926	22,040,601	21,638,105	21,708,195	28,024,500
Q30	88.47	89.50	88.66	89.24	89.36	88.49
GC content (%)	54.51	55.03	55.81	52.15	53.00	52.75
Total reads	41,155,984	44,071,852	44,081,202	43,276,210	43,416,390	56,049,000
Mapped reads	27,032,277 (65.68%)	29,254,170 (66.38%)	28,955,373 (65.69%)	32,513,302 (75.13%)	32,261,454 (74.31%)	41,110,731 (73.35%)
Unique mapped reads	26,040,780 (63.27%)	28,099,343 (63.76%)	27,866,465 (63.22%)	31,764,931 (73.40%)	31,549,418 (72.67%)	40,123,163 (71.59%)
Multiple map reads	991,497 (2.41%)	1,154,827 (2.62%)	1,088,908 (2.47%)	748,371 (1.73%)	712,036 (1.64%)	987,568 (1.76%)
Reads map to ‘+’	13,233,281 (32.15%)	14,285,546 (32.41%)	14,166,568 (32.14%)	16,123,649 (37.26%)	16,011,892 (36.88%)	20,356,053 (36.32%)
Reads map to ‘−’	13,260,067 (32.22%)	14,297,503 (32.44%)	14,166,223 (32.14%)	16,093,216 (37.19%)	15,979,783 (36.81%)	20,336,161 (36.28%)

Clean reads = read numbers of single-ended sequencing (150 bp). Total reads = read numbers of double-ended sequencing. Total mapped% = total mapped/clean reads; total mapped: clean reads mapped on the sheep genome. Unique mapped% = unique mapped/total mapped; unique mapped: reads mapped only one region to the reference genome. Multiple mapped% = multiple mapped/total mapped; multiple mapped: reads mapped more than one region to the reference genome.

**Figure 1:**
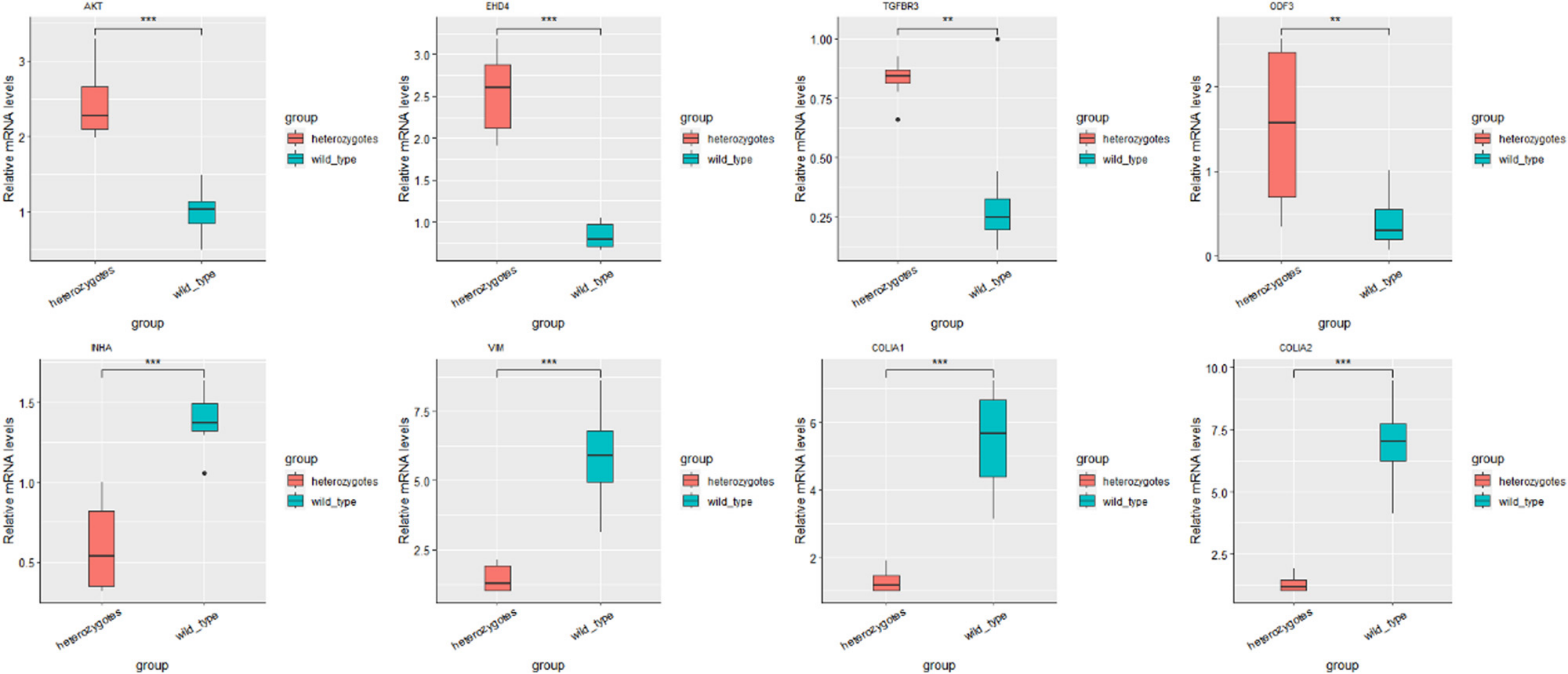
Differentially expressed mRNAs were examined in different genotype testis tissue. One asterisk (*P* value <0.05) indicates a significant difference between two groups, two asterisks (*P* value <0.01) indicate a possible highly significant difference between two groups, and three asterisks (*P* value <0.001) indicate a highly significant difference between two groups.

**Figure 2:**
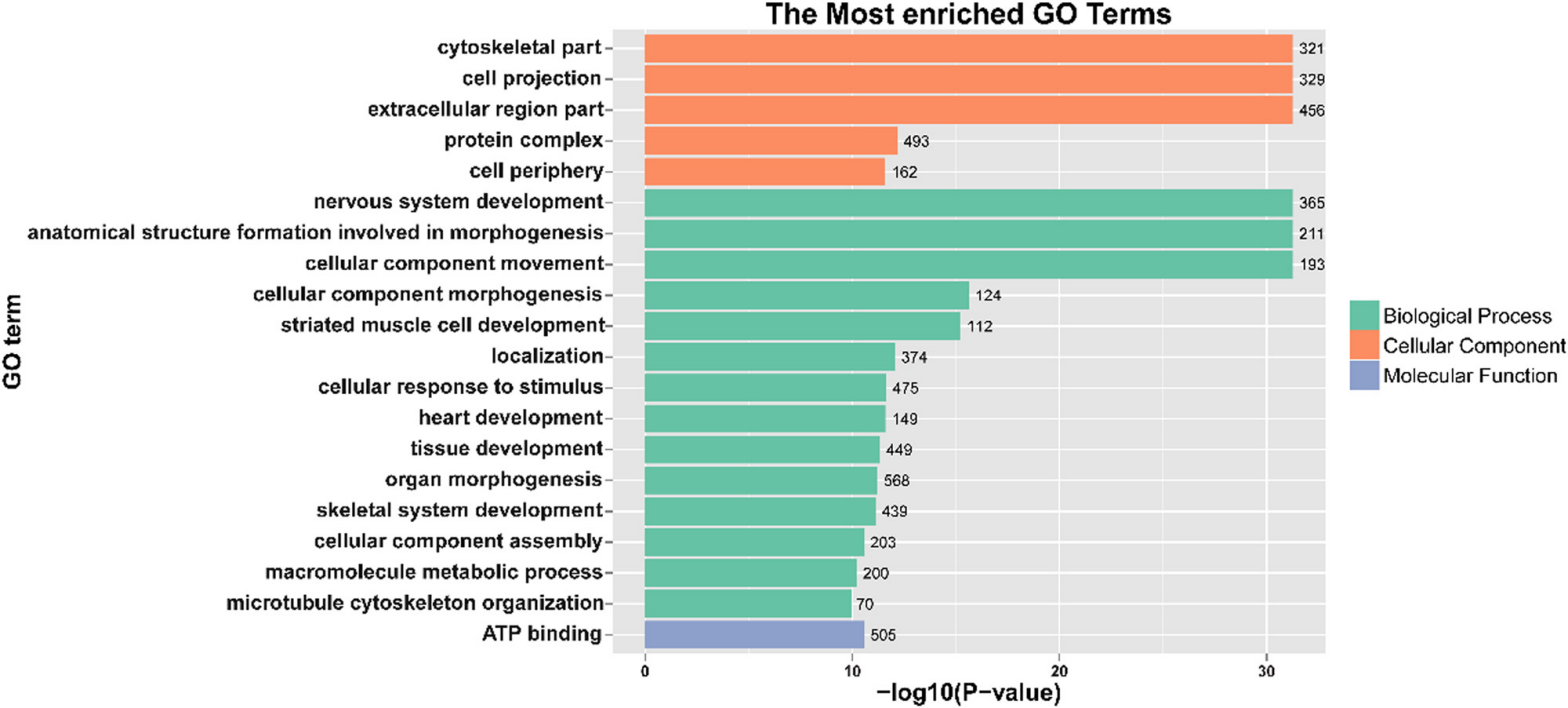
GO enrichment analysis of DEGs in wild type vs heterozygous group. BP: biological process, MF: molecular function, and CC: cellular component.

Sequencing analysis revealed that *AKT*, *EHD4, TGFBR3*, *ODF3*, *INHA*, *VIM*, *COLIA*1, and *COLIA2* were DE in the testes of different genotypes. An extensive literature research revealed that these genes are closely related to testicular development, sperm morphology, and spermatogenesis. Therefore, we selected these genes for qPCR validation. To confirm the gene expression pattern identified by RNA sequencing, eight genes including four up- (*AKT*, *EHD4*, *TGFBR3*, *ODF3*) and four downregulated (*INHA*, *VIM*, *COLIA1*, *COLIA2*) genes were selected for real-time RT-PCR using testis samples from different genotypes. The results showed that the expression patterns of eight genes were consistent with those of the RNA sequencing results (Figure 1), implying that our RNA-seq results and analysis methods were highly reliable. GO enrichment analyses showed that the DEGs were significantly enriched for GO terms involving cell morphogenesis, tissue development, and spermatogenesis (Figure 2).

### 3.2 miRNA expression analysis from testes of different genotypes

**Figure 3:**
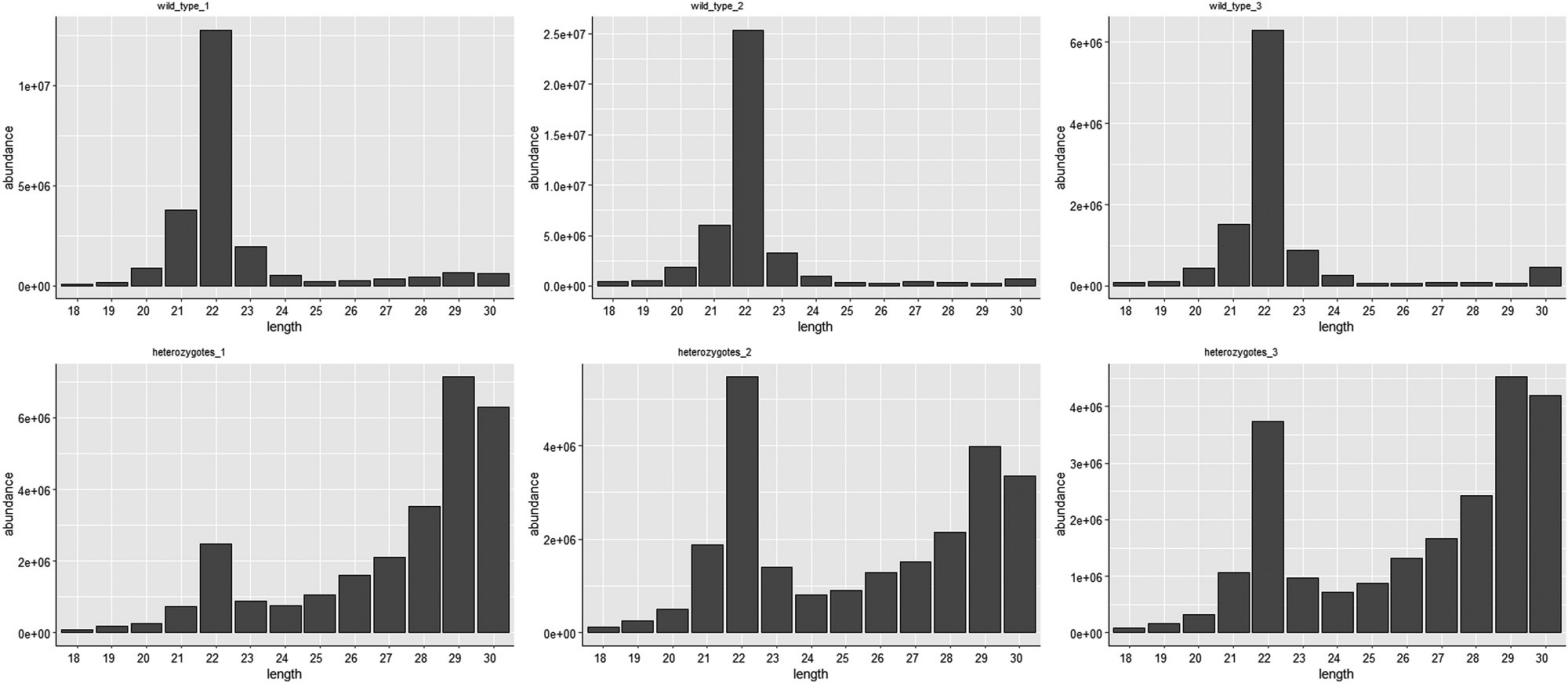
Length distribution and abundance of sequences in each library in miRNA analysis.

The Q30 values of the wild type and heterozygotes were more than 97.17%. The read length demonstrated a bimodal with 21–23 and 26–32 nt in six libraries (Figure 3), which was consistent with the classical fragments of miRNAs. These results showed that the sequencing quality was feasible. After obtaining clean reads, the clean reads were categorized using some database. Subsequently, unannotated clean reads of sRNA were mapped to the sheep reference genome (Ovis_aries3.1). Approximately 58.74–65.55% of clean reads were mapped to the sheep reference genome (Ovis_aries3.1) (Table 3). The results indicated that the diversity and distribution of miRNAs in testes samples from different genotypes, including wild type and heterozygous, are relatively abundant. These results suggest that miRNAs are involved in testicular development. Finally, 243 DE miRNAs, including 158 up- and 85 downregulated miRNAs, were found in the wild type compared to the heterozygote group.

**Table 3:** Proportion of sRNA unannotated clean reads mapped to reference genome

Sample	Group	Total_reads	Mapped_reads	Percentage	Mapped_reads (+)	Percentage	Mapped_reads (-)	Percentage
SRWT1	Wide type	22,349,308	13,127,773	58.74	9,489,962	42.46	3,637,811	16.28
SRWT2	Wide type	38,961,364	25,407,601	65.21	19,440,026	49.90	5,967,575	15.32
SRWT3	Wide type	9,818,354	6,435,474	65.55	4,801,936	48.91	1,633,538	16.64
SRHT1	Heterozygote	26,304,263	15,124,368	57.50	7,559,945	28.74	7,564,423	28.76
SRHT2	Heterozygote	22,876,684	14,065,416	61.48	7,931,266	34.67	6,134,150	26.81
SRHT3	Heterozygote	21,153,623	12,620,989	59.66	6,866,318	32.46	5,754,671	27.20

### 3.3 Integrated function analysis of genes and miRNA expression

**Figure 4:**
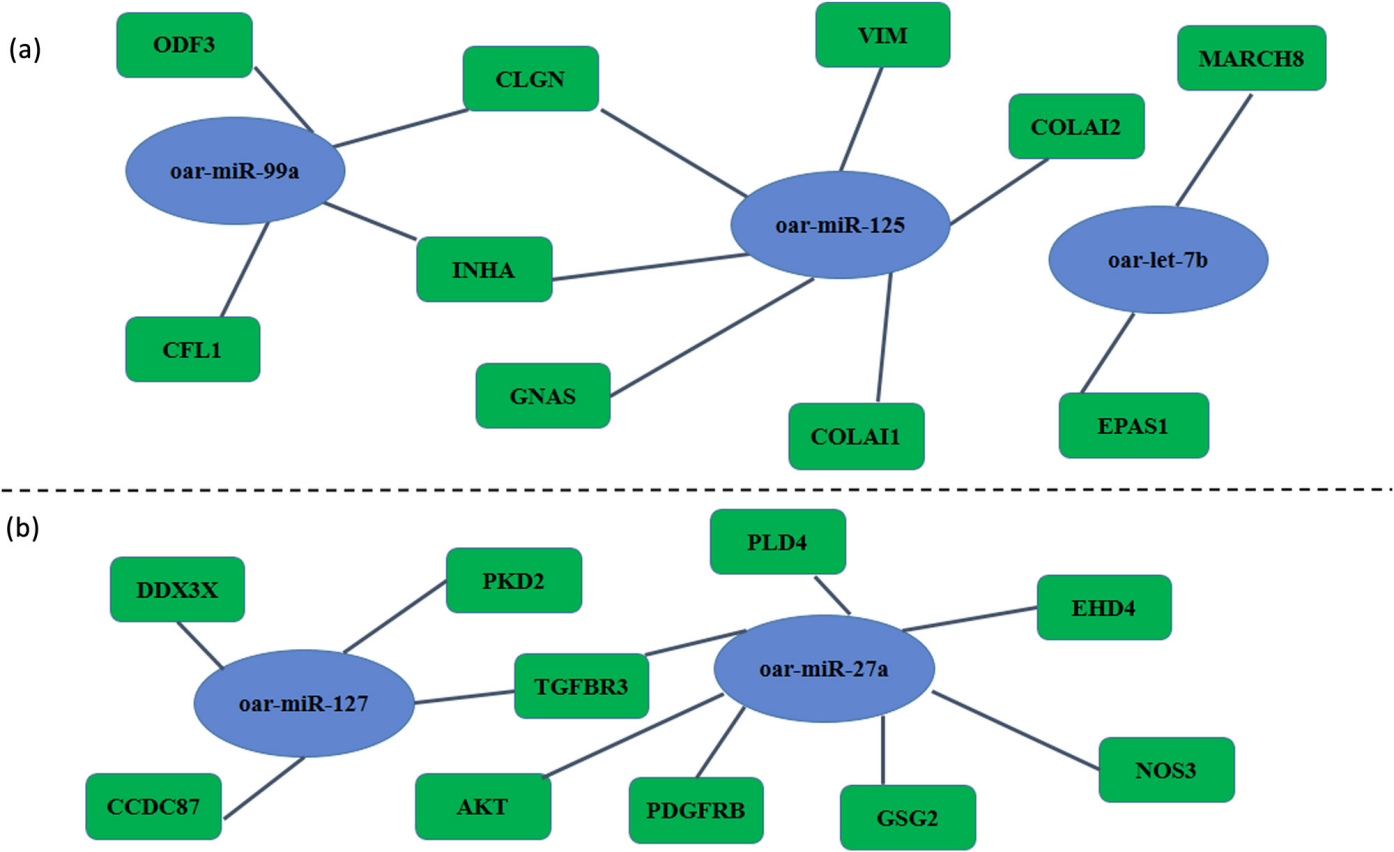
Regulatory network mRNA interaction miRNA; (a) represents Type I, where up-regulated mRNA interacts with down-regulated miRNA in wild type compared to the heterozygous group, (b) represents Type II, where down-regulated mRNA interacts with up-regulated miRNA in wild type compared to the heterozygous group.Blue labels represent miRNA and green labels represent mRNA.

Compared to the heterozygote group, 48 TDETGs were observed in the wild type, including 16 upregulated TDETGs corresponding to 8 downregulated miRNAs and 32 downregulated TDETGs corresponding to 12 upregulated miRNAs (Table 4). miRNAs and protein-coding genes are involved in regulatory networks to regulate spermatogenesis and testes development. In terms of miRNAs, which can positively and negatively regulate target genes, both types of networks were constructed. Type I belongs to the regulatory network of upregulated mRNAs interacting with downregulated miRNAs in wild type compared to heterozygote group. Type II belongs to the regulatory network of downregulated mRNAs interacting with upregulated miRNAs in the wild type compared to the heterozygote group (Figure 4). A total of three miRNAs and ten genes were present in the type I network, whereas two miRNAs and ten genes were present in the type II network. We found that oar-miR-27a, miR-125b, miR-127, *EHD4*, *TGFBR3*, and *PDGF* were all active in the regulatory network.

**Table 4:** Typical differential expression miRNAs and target genes involving testicular development, including positive and negative regulation in wild type compared to heterozygote group

High expression of miRNA	Low expression of target genes	Lower expression of miRNA	Higher expression of target genes
oar-miR-27a	*EHD*4;*PDGFRB*; *AKT*;*GSG*2;*NOS*3;*PLD*4;*TGFBR*3	oar-miR-99a	*ODF3;CFL1;CLGN;INHA*
oar-miR-127	*PKD*2;*DDX3*X;*CCDC*87;*TGFBR*3;*AKT*	novel-64	*NSUN7;ANKRD31;MARCH8;GSG2*
novel-55	*SPN*;*MAP3K*8	oar-let-7b	*MARCH8;EPAS1*
novel-28	*SH3PXD2*B;*HR*;*RBCK*1;*STARD*13;*BCL9L*;*IFI35*;*PDGFRB*	oar-miR-125a	*VIM;COLAAI1;COLAI2;GNAS;INHA*
novel-18	*NBL*1;LOC101109421	novel-40	*ENSOARG00000001264*
novel-30	*IGFN*1;*NFKB*;*IL4R*;*VWF*;*DOCK1*	novel-62	*ESPL1;CCDC87*
novel-45	*KDELC2*;*PTPN21*	novel-45	*PPP2R4;EHD4*
novel-89	*SARDH*	novel-68	*KIAA1328;TGFBR3*
novel-56	*WDR*45		
novel-28	*CD*248;*FBLN*2		
novel-19	*NBL*1; *PDGFRB*		
novel-59	*STARD*13		

## 4 Discussion

Xizang sheep, also known as Tibetan sheep, are widespread in QTP and adjacent areas. Using molecular markers of the FecB gene, the core population of multiparous Tibetan sheep was established and the technical problems of low fecundity in ewe were solved. However, ram fertility is poorly researched. Therefore, we investigated testicular genetics in wild-type rams and heterozygote rams to find effective molecular markers for measuring ram fertility.

Sequencing results revealed a big difference in the wild type compared to the heterozygote group. In general, clean reads were assigned to three major regions, including exonic, intergenic, and intronic. Approximately 65.68–74.72% of clean reads were mapped to the sheep reference genome, of which 63.27–73.03% were uniquely mapped. The efficiency of mapping to the reference genome was low and we give several possible reasons for this. First, the Tibetan sheep selected are breed specific and may differ from the reference genome. Second, the sheep reference genome does not contain the Y chromosome, and we sequenced testicular tissue. A total of 2,034 up- and 1,876 downregulated genes were identified in the wild type compared to the heterozygote group. These genes may be important for testicular growth and spermatogenesis.

The length distribution of small RNAs was concentrated in the two peaks of 19–24 and 26–32 nt. Notably, the clean reads of the wild-type group were mainly miRNA based. Interestingly, the miRNA level gradually decreased, and the level of piRNA gradually increased in the heterozygote testes; these data were coherent with previous descriptions. These results indicate that the regulation of spermatogenesis is complex. Consistently, Hirano et al. [39] performed small RNA-seq analysis in the marmoset and found that piRNA is dominant in adult testes. Li et al. used high-throughput sequencing to compare miRNA expression in immature and mature pigs and reported that the proportion of miRNAs was higher before sexual maturity in large white pigs, but not in Luchuan pigs [40]. Lian et al. found a significant difference in the proportion of small RNAs before and after sexual maturation [41]. Similar to the results of this experiment, the proportion of miRNAs in the wild-type group was significantly higher than that in the heterozygote group. These results suggest that both miRNA and piRNA may be involved in testicular development during sexual maturity.

TDETGs were used in this sequencing study to identify potential regulatory factors that influence testicular development. A total of 20 potential miRNAs were identified, including 8 down- and 12 upregulated miRNAs. These miRNAs include five known miRNAs (oar-miR-27a, oar-let-7b, oar-miR-125b, oar-miR-99a, and oar-miR-127), and newly discovered miRNAs. The functions of some known miRNAs have been reported. miR-27a is a differentiation marker for spermatogonial stem cells and is highly expressed in dairy goat testis cells [42]. In the mRNA–miRNA regulatory network constructed in this study, we screened miR-27a, suggesting that miR-27a may have potential regulatory effects on testicular development and differentiation of spermatogonial stem cells in sheep. Thus, these studies show that miRNA function is conserved in the vast majority of animals. In addition, studies in pigs have shown that miR-27a can be used as an internal reference gene for assessing the quality of cryopreserved sperm [43]. Studies have shown that miR-125b plays a crucial role in regulating zygote genome activation in oocytes and embryos [44]. miR-125b can target the degradation of the *PAP* gene, leading to an increase in testosterone concentration, a decrease in sperm count, and an increase in abnormal sperm ratio in male mice [45]. Related studies have shown that miR-125b is highly expressed in germ cells and directly regulates the secretion of testosterone by targeting PAP, increasing the mitochondrial DNA copy number in sperm cells, and ultimately affecting semen quality [45]. This indicates that miR-125b has a positive effect on reproductive performance and could be used as a drug and diagnostic target for male sterility. In the mRNA–miRNA regulatory network constructed in this study, we screened miR-125b, suggesting that miR-125b plays a key role in the development of sheep testes, especially hormone secretion and energy metabolism. miR-125b was found to play an important role in sheep reproduction. Although other miRNAs, such as miR-99 and miR-299, have been identified, their roles in reproduction are unknown [46,47]. Studies have shown that they can regulate stem cell self-renewal, ovarian cancer cells, and glioma, suggesting that their roles in reproduction should not be neglected.

Furthermore, we also found that the miRNA target genes were involved in reproduction. For example, the target gene *EHD4* of miR-27a, which was originally described as a protein expressed in the extracellular matrix, has been shown to be a cytosolic and endosomal vesicle-associated protein [48]. George et al. [26] found that although *Ehd4* ^−/−^ mice were fertile male, their 31 days postpartum testicular weight decreased by 50%, and apoptosis gradually increased. Other defects, including reduced seminiferous tubule diameter, abnormal regulation of spermatogenic epithelium, and abnormal head of slender sperm cells, are also present in *Ehd4* ^−/−^ mice, which eventually result in lower sperm counts and lower fertility. In adult testes, *EHD4* is highly expressed in primary spermatocytes and *EHD4* deletion alters the levels of other EHD proteins in an age-dependent manner. The results suggest that *EHD4* plays a role in the normal development of mitosis and late germ cells, and that EHD protein-mediated endocytic cycling is important for germ cell development and testicular function [26]. The target gene *TGFBR3* is regulated by miR-127 and miR-27a. Beta glycans (*TGFBR3*) belong to one of the ligands of the transforming growth factor beta (*TGFβ*) superfamily. Sarraj et al. [49] found that *Tgfbr3* ^−/−^ mice developed severe fine cords at 12.5–13.5 days after conception and their fetal stromal cell function was affected; their experimental data show that *TGFBR3* is necessary for the normal formation of the spermatic cord, the development of fetal stromal cells, and the establishment of endocrine function. *TGFBR3* was also screened in the network constructed in this study, suggesting that this gene is important for fine testicular formation, interstitial cell development, and endocrine function in sheep. The findings confirmed that miR-27a and miR-127 potentially control testis size. Testicular development is controlled by complex levels of gene regulatory proteins, growth factors, cell adhesion molecules, signaling molecules, and hormones that interact with each other through controlled relationships, usually in a short period of time. Platelet-derived growth factor (*PDGF*) is a key regulator of connective tissue cells during embryogenesis and pathogenesis, including *PDGFA*, *PDGFB*, *PDGFRA*, and *PDGFRB*. The results of this study suggested that *PDGFRB* is a target of miR-27a. Mice with mutations in *PDGF* had normal testicular development before birth, normal fetal cytoplasmic cell numbers, and normal masculine functions. However, after birth, the gene mutation caused testicular shrinkage, loss of mesenchymal cells, decreased testosterone, and impaired spermatogenesis [50]. Another interesting finding is regulation of the target genes *COLIA1* and *COLIA2* by miR-125. There is evidence that *COLIA1* and *COLIA2* are involved in type A spermatogonia and play a role in mediating the isolation and migration of germ cells during spermatogenesis [51]. The aforementioned evidence revealed that these genes may play a key role in regulating testicular development.

## 5 Conclusions

In this study, 20 potential miRNAs, including 8 down- and 12 upregulated miRNAs were identified by constructing mRNA–miRNA regulatory networks. The miRNA target genes, namely, *TGFBR3*, *PDGFRB*, *Col1a2*, and *EHD4,* are related to reproduction. Therefore, these miRNA molecules can be used as potential candidate small molecules to regulate the testicular size and provide a theoretical reference for the genetic regulation of testicular development in sheep.
